# In Situ Self-Assembling Liver Spheroids with Synthetic Nanoscaffolds for Preclinical Drug Screening Applications

**DOI:** 10.1021/acsami.3c17384

**Published:** 2024-05-14

**Authors:** Lina Wu, Driton Vllasaliu, Qi Cui, Bahijja Tolulope Raimi-Abraham

**Affiliations:** King’s College London, Faculty of Life Sciences and Medicine, School of Cancer and Pharmaceutical Sciences, Institute of Pharmaceutical Science, Franklin-Wilkins Building, 150 Stamford Street, London SE1 9NH, U.K.

**Keywords:** liver, spheroids, nanofibre, scaffolds, drug-induced liver injuries

## Abstract

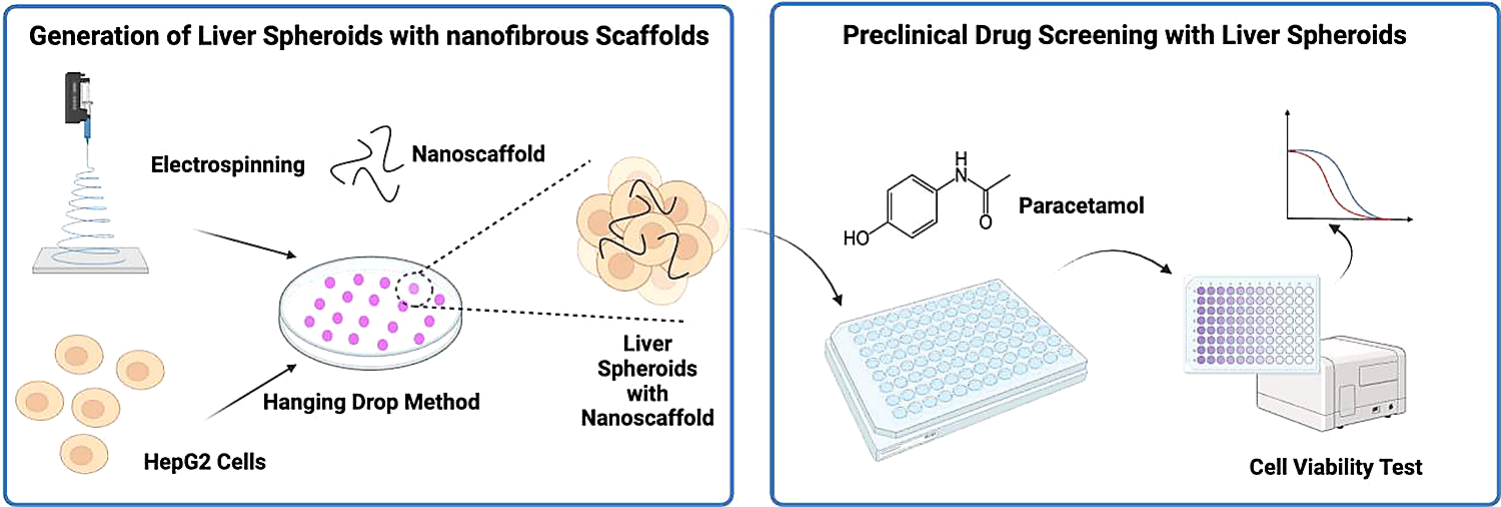

Drug-induced liver injury (DILI) is one of the most common reasons for acute liver failure and a major reason for the withdrawal of medications from the market. There is a growing need for advanced in vitro liver models that can effectively recapitulate hepatic function, offering a robust platform for preclinical drug screening applications. Here, we explore the potential of self-assembling liver spheroids in the presence of electrospun and cryomilled poly(caprolactone) (PCL) nanoscaffolds for use as a new preclinical drug screening tool. This study investigated the extent to which nanoscaffold concentration may have on spheroid size and viability and liver-specific biofunctionality. The efficacy of our model was further validated using a comprehensive dose-dependent acetaminophen toxicity protocol. Our findings show the strong potential of PCL-based nanoscaffolds to facilitate in situ self-assembly of liver spheroids with sizes under 350 μm. The presence of the PCL-based nanoscaffolds (0.005 and 0.01% w/v) improved spheroid viability and the secretion of critical liver-specific biomarkers, namely, albumin and urea. Liver spheroids with nanoscaffolds showed improved drug-metabolizing enzyme activity and greater sensitivity to acetaminophen compared to two-dimensional monolayer cultures and scaffold-free liver spheroids. These promising findings highlight the potential of our nanoscaffold-based liver spheroids as an in vitro liver model for drug-induced hepatotoxicity and drug screening.

## Introduction

In vivo and in vitro models are important tools in the field of pharmaceutical research. For decades, preclinical drug screening studies have relied heavily on experimental animal models.^1^ However, animal models always suffer from ethical concerns, limited tissue availability, high cost, and species-specific variations, which hinder the application.^2^ According to the US Food and Drug Administration (FDA) Modernization Act 2.0, the approval of new medicines no longer requires animal testing.^3^ This bill allows “alternatives to animal testing for purposes of drug and biological product applications”, which highlights the need for reliable nonanimal models, i.e., cell culture models.

Cell culture models allow cells from different sources to grow in physiological conditions with controllable laboratory methods and have been widely used in preclinical drug research, gene function, and disease mechanisms studies. Compared to animal models, cell-based models possess several advantages including lower cost, easier access to human-specific tissues, less ethical concerns, and the ability to conduct high-throughput screening for preclinical drug discovery.^4,5^ A range of cell-based platforms from two-dimensional (2D) monolayer cultures to more complicated three-dimensional (3D) structures such as organoids or spheroids have been employed as substitutes for animal models in biomedicine areas.^6^ However, monolayered 2D culture models will rapidly lose functionality and phenotypes and have a limited expression of drug-metabolizing enzymes, especially CYP 450.^7^ In contrast, 3D cell culture allows the cells to grow or interact with their surroundings in all three dimensions in vivo. As a result, unlike 2D culture cells, in which cells are equally exposed to nutrients and gas in the culture medium, in 3D culture systems, the cells are exposed to nutrients and gas in a gradient manner, thus cell behaviors including cell signaling, gene expression, and metabolism functions are more closely related to those in vivo.^8^ Therefore, 3D models are considered to be able to recapitulate the complex microenvironments in human tissues.^9,10^ Spheroids are spherical, self-assembled 3D cell aggregates. A major advantage of spheroids over other 3D cultured models, such as organoids, is that they are more cost-effective and the generation process is simpler and more rapid, which makes spheroids more suitable for high-throughput drug screening applications.^11,12^ Different techniques have been used to generate spheroids, including the hanging drop method, rotating bioreactors, ultralow-attachment plates (ULA), and liquid overlay.^13^ Compared with other techniques, the hanging drop method has several advantages such as (i) does not require special equipment and is more cost-effective than commercially used ULA plates; (ii) it can be used to coculture different cell lines; (iii) the formation of the spheroids is rapid and the size of spheroids can be controlled easily.^14^ In the hanging drop method, the cells accumulate at the bottom of the droplet under gravitational force and form a single spheroid.^15^ The hanging drop method has been used to generate human liver spheroids as in vitro models for the prediction of drug hepatotoxicity and in vitro drug testing and has been shown to have enhanced liver-specific functions and higher sensitivity to drug treatment than 2D cell culture models.^16,17^

In vivo, cells grow within the extracellular matrix (ECM) and the cell/ECM interaction is of great importance for cellular functions since ECM provides structural support and controls the adhesion, proliferation, differentiation, and morphology of cells.^18^ Therefore, synthetic scaffolds that could mimic the function of natural ECM are considered to have the potential to improve cell behavior in 3D cell models.^19^ Nanofibers generated by electrospinning from synthetic polymeric materials such as polycaprolactone (PCL), poly(vinyl alcohol) (PVA), and poly(lactic-*co*-glycolic acid) (PLGA) have been used as scaffolds in a lot of 3D cell models. Similar to the natural ECM, the electrospun nanofibers can also offer a nanoscale porous structure and thus could enhance the efficiency of cell spheroid formation.^20^

However, nanofiber-based scaffolds used in most studies are in the form of nanofibre mats^21^ or sponges.^22^ In this way, it is difficult to create spheroids with a uniform size and shape. Besides, spheroids could not be separated and collected at a single spheroid level for further analysis and applications, such as high-throughput drug screening. Studies have shown that when transferred to the electrospun nanofibre membrane, the spheroids will lose the 3D structures within 7 days.^23^ Therefore, the development of nanoscaffolds that can be integrated into single spheroids is highly desired.

The liver is the major site of drug metabolism and detoxification due to the presence of metabolizing enzymes. As a result, the liver is particularly vulnerable to drug-induced liver injuries (DILI) especially when exposed to high drug concentrations and their metabolites.^24^ DILI can severely impair liver functionality and continues to be a major source of clinical attrition, precautionary warnings, and postmarket withdrawal of drugs.^25^ Liver toxicity (i.e., hepatoxicity) is also a common issue that can limit the clinical use of many drugs.^26^ The prediction of hepatoxicity and the elimination of drug candidates with an elevated risk of causing DILI early in the early drug development is important.^27^ Therefore, creating robust in vitro liver models that could reliably evaluate hepatotoxicity at the preclinical stage is of great importance.

Here, we generated self-assembled liver spheroids using synthetic electrospun PCL nanoscaffolds for preclinical screening applications. Primary human hepatocytes (PHH) are the gold standard for drug metabolism and hepatotoxicity studies.^28^ However, the use of PHH still faces challenges such as the limited life span and availability, cellular dedifferentiation during in vitro culture, donor-to-donor variability, and high cost.^29^ Hepatoma cell lines are considered to be good substitutes for primary cells in drug screening applications. HepG2 cells are the most widely used human hepatoma cell line in drug metabolism and hepatotoxicity studies.^30,31^ The cell line was isolated from hepatocellular carcinoma of a 15-year-old Caucasian male. They exhibit a nontumorigenic property with high proliferation rates and perform many differentiated hepatic functions.^32^ While HepG2 cells may not fully replicate all functions of PHH, they do offer a valuable alternative for conducting initial investigations due to an unlimited life span, low cost, good availability, and high reproducibility of data.^33^ However, in conventional 2D cultures, HepG2 cells suffer from the limited expression of drug-metabolizing enzymes, especially cytochrome P450 (CYP450),^34^ which is an important determinant of the pharmacokinetics and toxicity of drugs. Poor expression of CYP450 and other metabolic enzymes in hepatocyte cell lines can be a key contributing factor in poor hepatoxicity prediction in humans. 2D cell cultured HepG2 cells have been shown to have poor liver function biomimicry such as low albumin and urea secretion hindering their application in preclinical screening.^33^ 3D HepG2 spheroids have shown enhanced liver functions.^35^ However, the prolonged culture of these spheroids is hindered by reduced cell viability due to central hypoxia, posing a significant challenge to the practical application of HepG2 spheroids.^36^

In our work, we explored the use of short electrospun PCL nanofibers, which we refer to as nanoscaffolds, as synthetic ECM to facilitate in situ self-assembly of HepG2 liver spheroids via the hanging drop method to improve biomimicry, spheroid viability, and minimize central hypoxia and necrosis. The aim of this study is to compare the behavior of spheroids with and without nanoscaffolds to emphasize the role of nanoscaffolds in the system. Additionally, the work explores the influence of nanoscaffold concentration on spheroid formation and spheroid properties. First, we compared the behavior of cells cultured in the traditional 2D monolayer format with those grown as 3D HepG2 spheroids, with and without nanoscaffolds (at different concentrations). Second, we assessed the impact of our nanoscaffolds on cell behavior, spheroid cell viability, biofunctionality (namely albumin and urea secretion), and drug metabolism function (drug-metabolizing enzyme activity and acetaminophen metabolism).

We proved that the intergradation of the nanoscaffolds could enhance the viability and liver-specific functionality of liver spheroids, providing a promising platform for drug screening and tissue engineering.

## Results and Discussion

### Characterization of Polycaprolactone (PCL) Nanoscaffolds

Electrospinning was used here because it can generate nanoscale fibers with a high surface-to-volume ratio and a porous network structure similar to the native ECM.^37^ In this study, we used PCL, a well-known semicrystalline and biodegradable polymer commonly used in cell culture and tissue engineering. PCL was used in this work because compared with other synthetic and natural polymers, it possesses several desirable properties including low-cost, biodegradable, biocompatible, bioresorbable, and satisfactory mechanical characteristics, such as a slow degradation rate (2–4 years depending on the starting molecular weight) and higher elastic modulus.^38^ Several studies have shown that PCL-based scaffolds improve cell attachment, proliferation, and differentiation compared with scaffold-free spheroids.^39^

**Figure 1:**
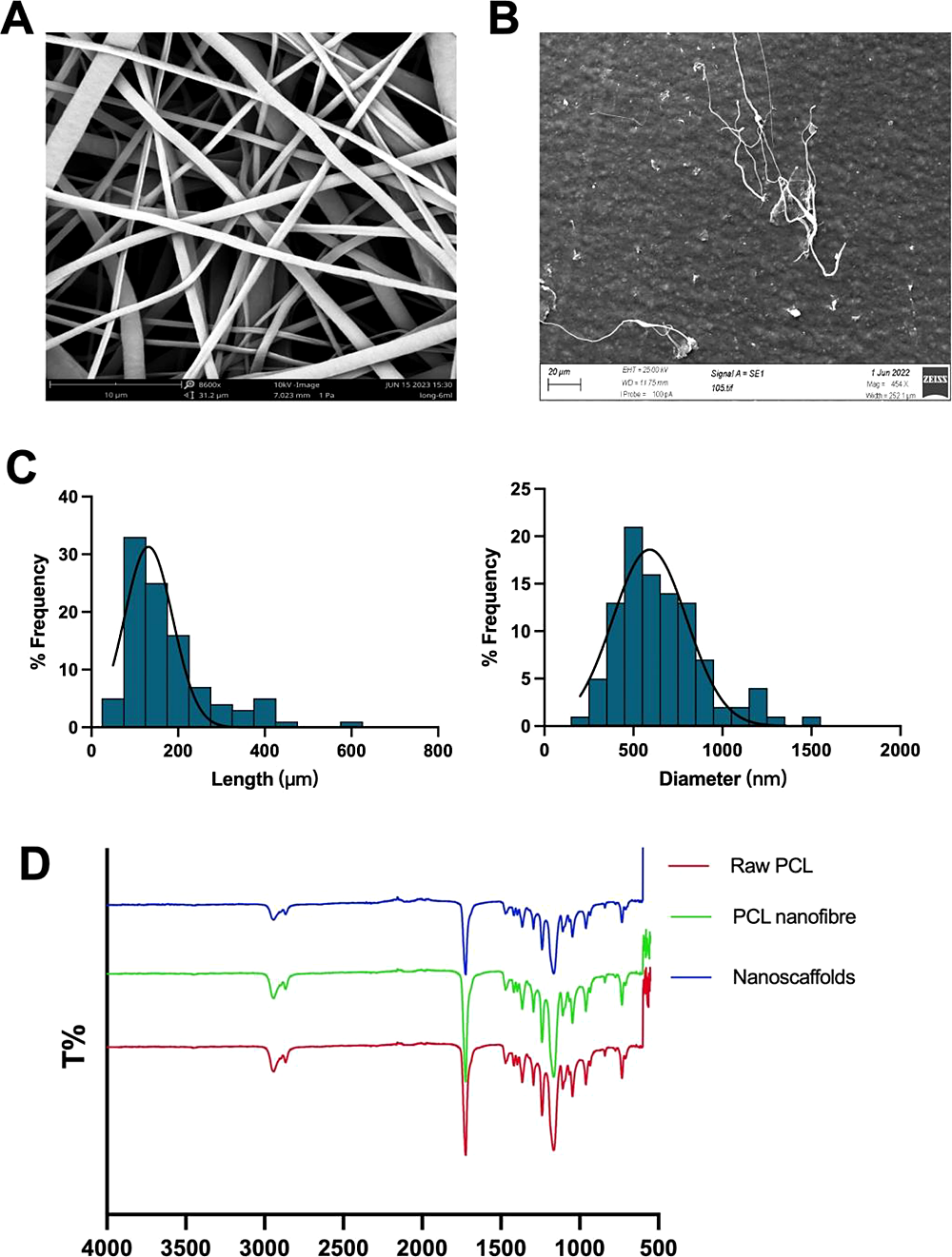
Characterization of nanofibers. (A) SEM images for PCL nanofiber mesh. (B) SEM images for nanoscaffolds. (C) Distribution of the length and diameter of the nanoscaffolds (Taken from 100 different nanofibers). (D) ATR-FTIR for Raw PCL, PCL nanofibre mesh and nanoscaffolds.

In our work, continuously electrospun PCL nanofibers were successfully generated with average diameters of 633.73 ± 126.02 nm (Figure 1A) and a porosity of 61.6 ± 3.5%, which is considered to be suitable for the scaffold of cells (60–70%).^40^ Nanofibre porosity influences its wettability, with higher porosity resulting in increased wettability, which in turn influences the ability of cells to adhere and proliferate on its surface.^41^ Nanofibre diameter is known to influence cell attachment and proliferation.^21,42^ Several studies have shown that cells prefer to adhere to nanofibers with smaller diameters (around 500 nm).^42−44^ Therefore, the diameters of the nanoscaffolds in our study were considered suitable for cell adherence.

The fibrous structure of the nanoscaffolds was preserved after cyrogrinding (Figure 1B), indicating that the grinding process did not destroy the morphological properties of the nanofibre. The average diameter and length of nanoscaffolds were 651.2 ± 243.9 nm and 175.8 ± 97.8 μm, respectively (Figure 1C, taken from 100 different nanofibers).

ATR-FTIR was conducted to define the chemical components of the nanoscaffolds. As shown in Figure 1D, in all the samples analyzed, distinct characteristic bands of PCL were observed. Specifically, the carbonyl groups associated with the ester bonds exhibited a band at 1724 cm^–1^. CH2 stretching was evident at around 2860 and 2940 cm^–1^. Additionally, the C–O symmetric stretching band peak was observed at approximately 1167 cm^–1^. The spectra obtained for PCL nanofiber mesh and nanoscaffolds were almost identical to the ones obtained for pure PCL raw material, which confirmed that the chemical properties were not changed after going through the electrospinning and cryogrinding processes.

Furthermore, we observed that the presence of PCL nanofibers could promote the proliferation of HepG2 cells in 2D culture over the duration of 4 days, indicating the nontoxic nature of PCL nanofibers (Figure S1).

**Figure 2:**
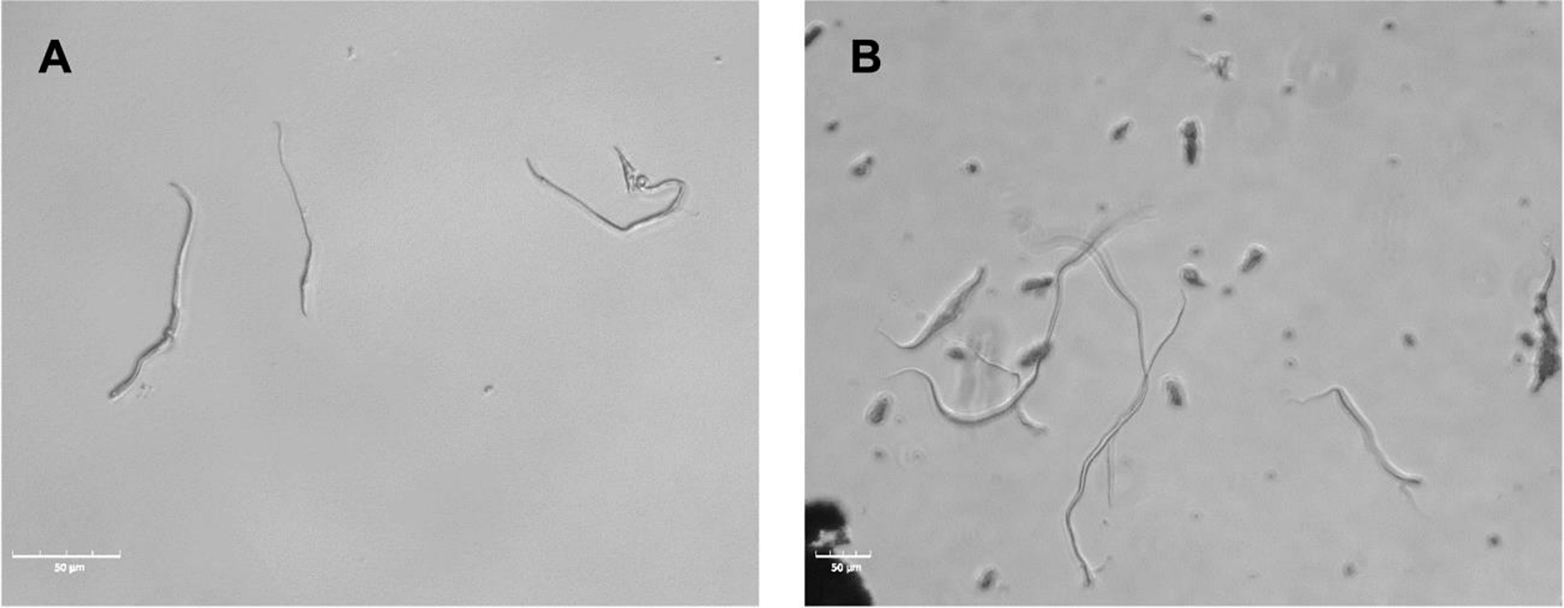
Phase-contrast microscope images of nanoscaffolds in cell culture medium on: (A) day 1 and (B) day 11. Scale bar: 50 μm. Objective: 10×.

To ensure that the PCL nanoscaffolds’ integrity was maintained during incorporation in the spheroid, the stability of the PCL nanoscaffolds in aqueous environments and their degradation behavior were investigated. The nanoscaffolds were immersed in a complete MEME cell culture medium for 11 days, which equates to the duration of the spheroid generation and drug treatment process. The nanoscaffolds maintained their fibrous morphology and remained intact in the cell culture medium throughout the 11-day experiment (Figure 2). This suggests that the nanoscaffolds could preserve their structural integrity in the cell culture medium over the experiment period, making them suitable scaffolds for the long-term culture of HepG2 spheroids.

### Characterization of HepG2 Spheroids with and without Nanoscaffolds

Studies have shown that the presence of synthetic nanofibers in spheroid formation results in the reduction of cell death due to cell nonadherence with spheroid formation promoted by the interaction of the cells with the nanofibers.^20^ Additionally, the benefits of synthetic nanofibers in spheroid formation have been highlighted including enhanced viability, maintenance of spheroid shape, prevention of hypoxia, apoptosis, and diffusion limitations.^45,46^ Studies employing short nanofibers have primarily focused on examining the impact of scaffold presence compared with scaffold-free models.^20,45,46^ However, there is a gap in the understanding of the influence of nanofibrous scaffold concentration on spheroid genesis and behaviors compared to scaffold-free models. To address this knowledge gap, we investigated the influence of the nanoscaffold concentration on the generation and behavior of spheroids. Our preliminary studies explored the influence of five nanoscaffold concentrations 0.2, 0.1, 0.02, 0.01, and 0.005% w/v on spheroid generation from day 1 to day 3 (i.e., spheroid genesis period) to ascertain which concentrations resulted in spheroid generation (Figure S2). Spheroid generation was not facilitated in the presence of 0.2, 0.1, and 0.02% w/v nanoscaffolds. However, spheroid generation occurred in the presence of 0.01 and 0.005% w/v nanoscaffolds. We observed that for spheroids successfully generated in the presence of nanoscaffolds, namely, at 0.01 and 0.005% w/v, the cells tended to adhere to the nanoscaffolds first and then aggregate to form the spheroid. In contrast, for spheroids generated without nanoscaffolds, the cells directly aggregate together. Further investigation is required in this area.

The work presented here was conducted at 0.01 and 0.005%. For ease, spheroids without nanoscaffolds and spheroids with 0.005 and 0.01% w/v nanoscaffolds will be referred to as S0, S-NS 005, and S-NS 01, respectively.

### HepG2 Spheroid Generation and Size Evaluation

**Figure 3:**
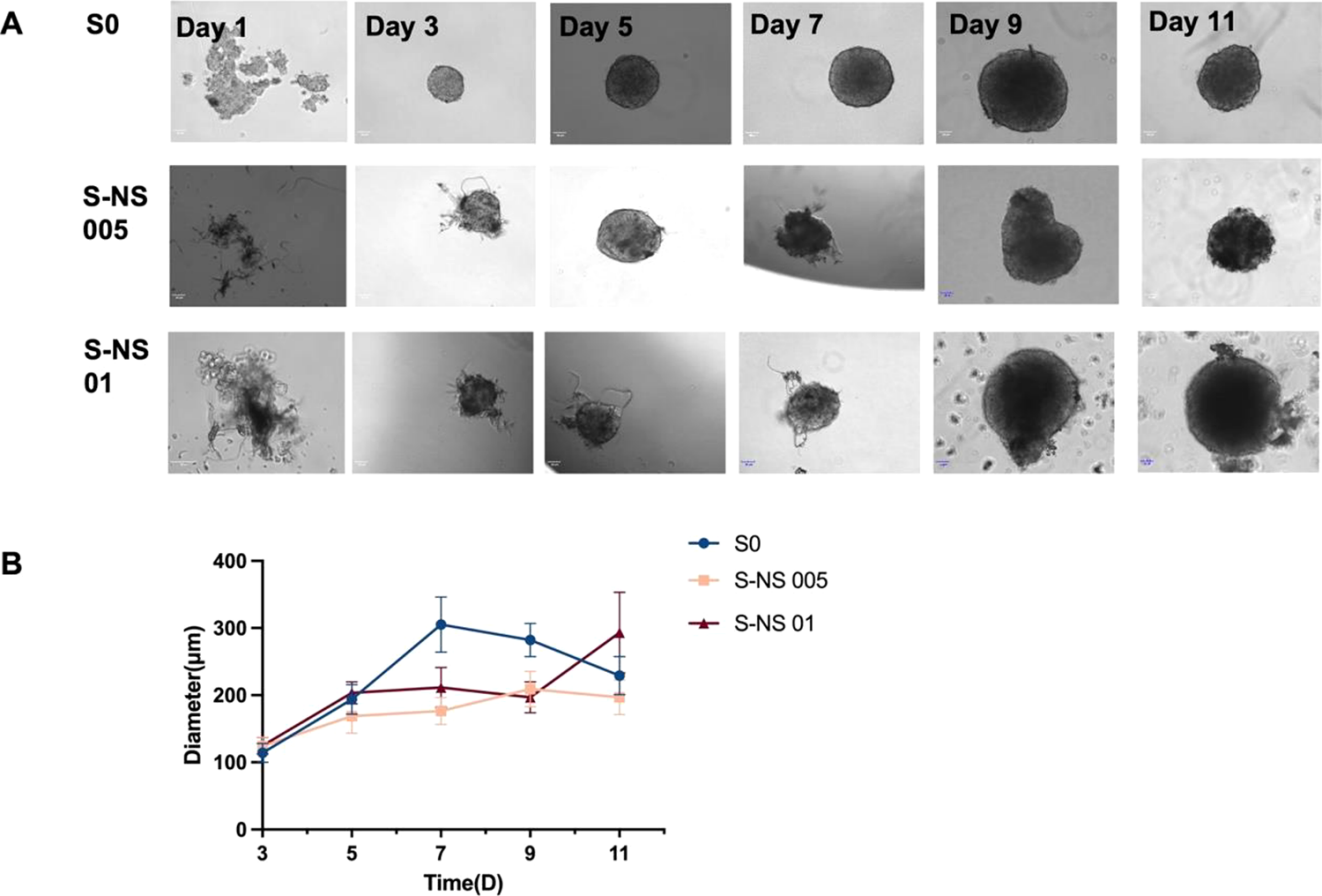
(A) Phase-contrast images of the generation process of spheroids over 11 days. Scale bar: 50 μm. (B) Growth curve of spheroids over 11 days. Spheroid diameter (μm) was plotted against culture time (days). Data are presented as mean ± standard error (*n* = 100 spheroids).

HepG2 spheroids with and without nanoscaffolds were successfully generated (Figure 3A). The process of our HepG2 spheroids involved loose and separated HepG2 cells, forming a compact cell aggregate with a spherical shape. As shown in Figure 3A, spheroids without scaffolds were formed by day 3. In the case of S-NS 005 and S-NS 01, cell aggregates were observed on day 3, although they evolved into more well-defined spherical and dense structures by day 5. This finding suggested that the presence of scaffolds may influence cell behavior during spheroid formation. A possible reason is that when the cells are introduced into the culture system with scaffolds, they may have a preference for attaching to the nanoscaffolds rather than establishing direct cell–cell contacts.^47,48^ This could be due to the physical properties of the PCL scaffolds, such as their surface characteristics or topography, which may promote cell adhesion and anchoring.^49^ The scaffolds likely provide attachment points and a stable substrate for the cells, allowing them to adhere and spread onto the scaffold material.^20^ This initial attachment to the scaffolds may facilitate the aggregation of cells, leading to the formation of cellular aggregates or clusters. Each drop formed a single spheroid, and all spheroids maintained a consistent, uniform spherical shape over the 11-day culture period. This is envisaged to lead to consistent drug penetration and response to hepatotoxicity, thereby enhancing the efficacy and relevance of drug screening protocols.

The size of the spheroids is a key factor for their applications since the biological activity of spheroids is directly correlated with their diameter.^50,51^ Evidence shows that spheroids with small diameters would not have the required tissue level of physiological features due to the lack of cell–cell interactions. On the other hand, for spheroids with diameters between 200 and 500 μm, chemical gradients (e.g., of oxygen, nutrients, and catabolites) will occur, and a central secondary necrosis is generally generated when the spheroids are larger than 500 μm because of the limited oxygen and nutrient diffusion, which hinders the clinical applications.^50,51^ Therefore, spheroids should have the smallest size dispersion that is feasible and adequate diameters in order to achieve a uniform and meaningful level of biological characteristics.^52^ There is not an identified spheroid size that is ideal for toxicity testing and other assays. Small spheroids with a size of around 150 μm have been proven to exhibit 3D cell–cell and cell-matrix interactions and an altered expression profile as compared to 2D cultures.^50^

To determine the effect of nanoscaffolds on the spheroid size, we calculated the diameters of 20 spheroids were calculated. According to the growth curve of spheroids over 11 days (Figure 3B), S0 gradually increased in diameter from 114.25 ± 13.6 μm on day 3 to a peak diameter of 305.16 ± 41 μm on day 7. After day 7, the diameter of S0 decreased slightly to 229.1 ± 28.4 μm on day 11, likely due to an increase in the number of dead cells. S-NS 005 showed a similar trend, with the diameter increasing steadily from 124.33 ± 13.35 μm on day 3 to 209.21 ± 26.46 μm on day 9, followed by a decrease to 196.89 ± 25.78 μm on day 11. In contrast, S-NS 01 showed a different trend, with the diameter steadily increasing from 124.67 ± 12.62 to 293.16 ± 60.14 μm over the course of 11 days. For drug screening purposes, we aimed to control spheroid diameters at around 200 μm, so as not to induce hypoxic culture conditions while recovering tissue-level physiological properties.

### Nanoscaffold Dispersion within Spheroids

**Figure 4:**
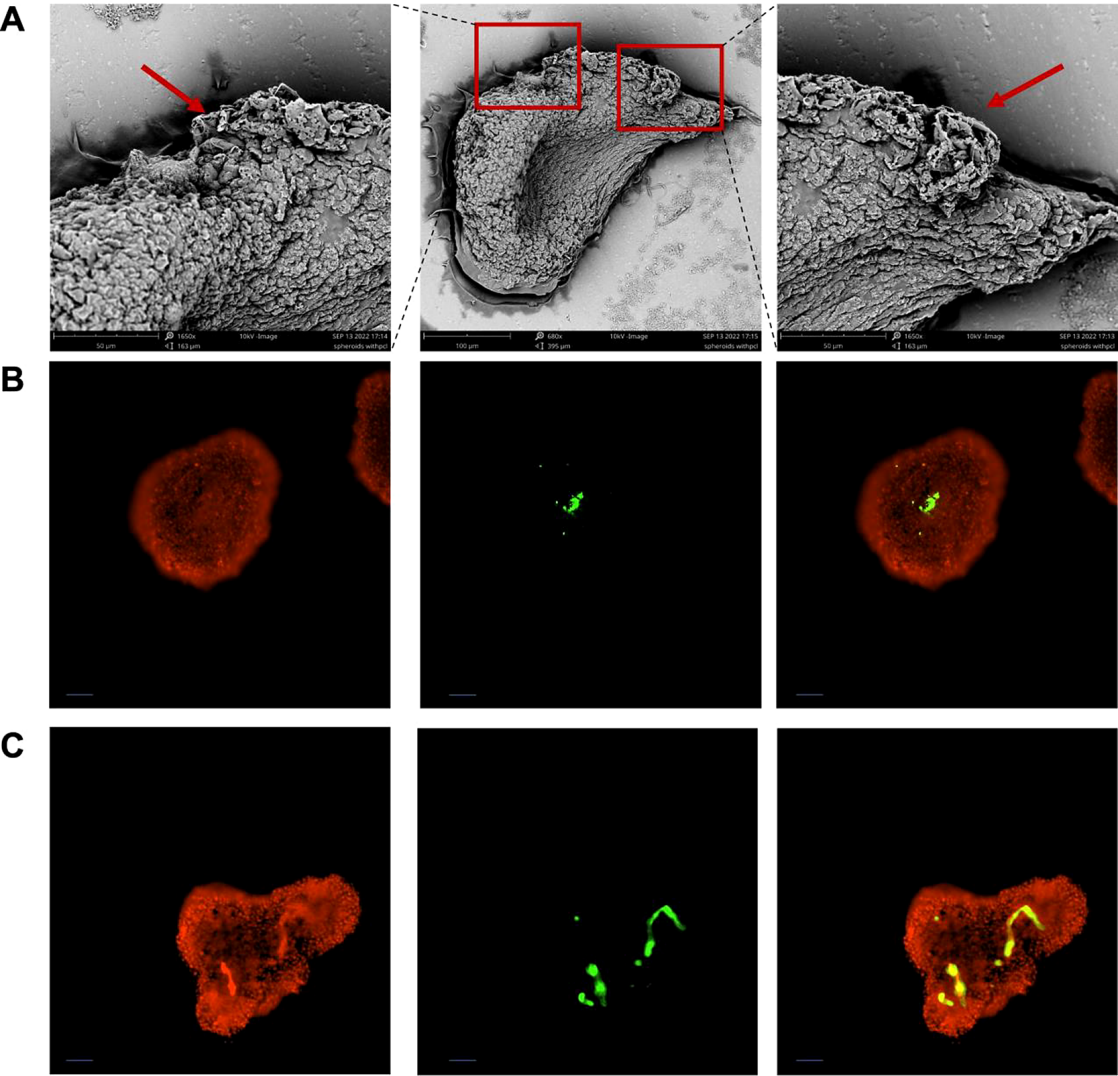
(A) SEM images of S-NS 005 on day 5; (B) S-NS 005 (C) S-NS 01 with HepG2 cells staining with Hoechst 33342(Red), coumarin-loaded PCL nanofiber fragments (Green) and overlay (Yellow). Scale bar: 50 μm. Objective: 10×.

To confirm the distribution of nanoscaffolds in spheroids, we conducted SEM analysis and fluorescent staining on S-NS 005 on day 5. SEM images (Figure 4A) show that nanofibers were observed at the edge of spheroids (red arrows) and exhibited tight cell–cell contact with engulfed fiber fragments. Fluorescent staining of cells and nanoscaffolds was conducted to further determine the distribution of nanoscaffolds in spheroids. S-NS 005 and S-NS 01 after 5 days of culture were stained with Hoechst 33342, with the color adjusted to red for clear overlay imaging. Coumarin-6 was encapsulated in fiber fragments by electrospinning. The overlaid fluorescence images (yellow) of Hoechst 33342 and coumarin-loaded fiber showed the distribution of PCL nanoscaffold aggregates in the central area of the spheroids (Figure 4B,C). In addition, we found that the presence of nanoscaffolds would affect the shape of the spheroids (more irregular shapes with S-NS 01). As a result, we chose the S-NS 005 samples for the drug screening applications as the S-NS 005 samples have higher uniformity.

### Impact of Nanoscaffolds on HepG2 Spheroid Cell Viability

**Figure 5:**
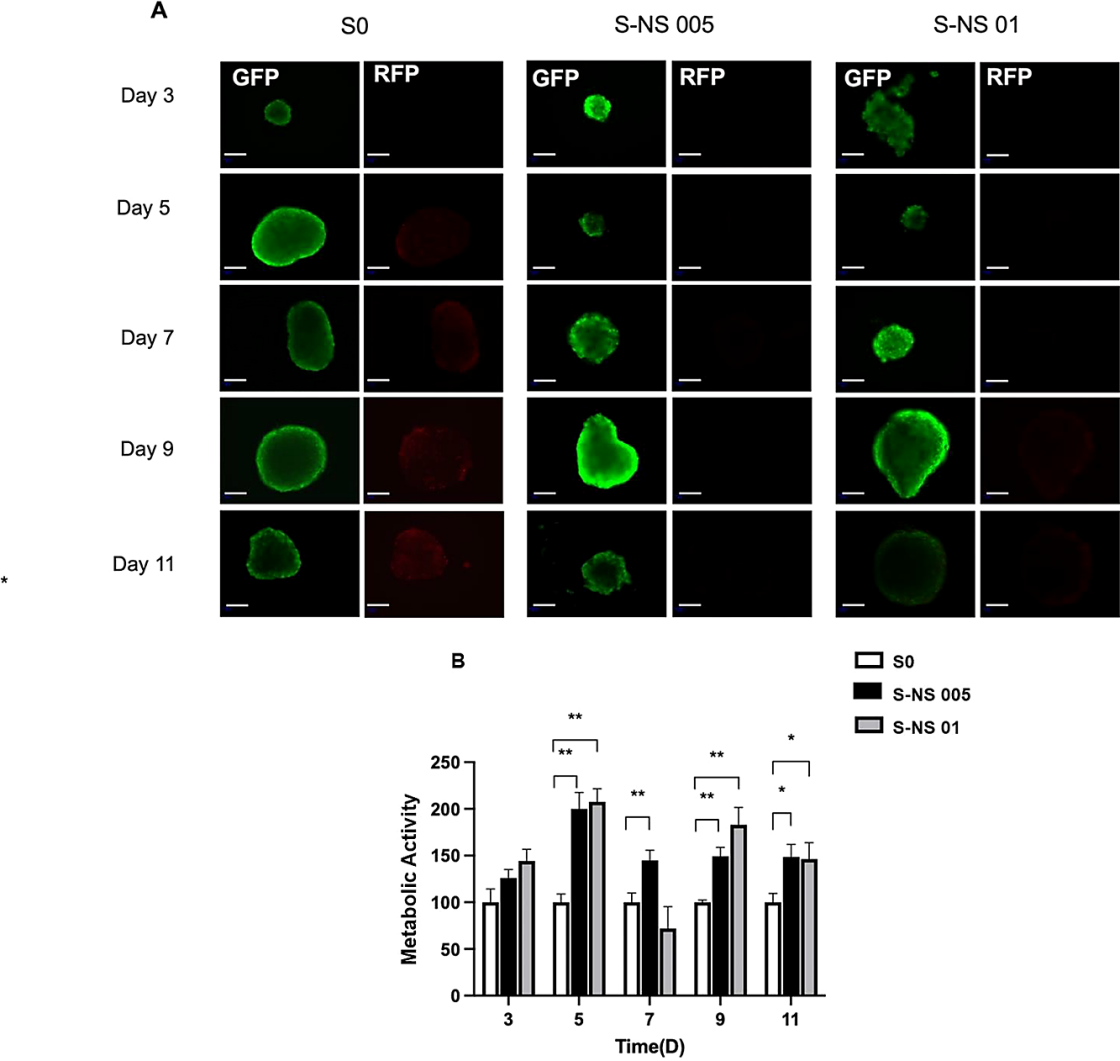
Cell viability of spheroids over the duration of 11 days. (A) live–dead staining images, live cells (GFP, green), and dead cells (RFP, red); Scale bar: 100 μm. Objective: 10×; (B) Cell metabolic activity based on ATP level assay. Data are presented as mean ± standard error (*n* = 3, * *p* < 0.01).

It is important for in vitro models to maintain long-term viability and preservation of relevant cellular phenotypes for predicting drug toxicity, especially in the study of chronic toxicity.^53^ The cell viability of spheroids, with and without nanoscaffolds, was evaluated by using the live–dead staining method. As shown in Figure 5A, a small number of dead cells (red) were observed in S0 from day 5, and the number of dead cells steadily increased until day 11, which was possibly due to the development of oxygen and nutrition gradients as the spheroid size increased. In contrast, cell viability improved in S-NS 005, with cell death occurring from day 7 and weaker fluorescence intensity of dead cells. A similar result was observed in S-NS 01, indicating enhanced cell viability throughout the 11-day culture period. Furthermore, spheroids could retain their original shape without any significant deformations or disruptions after the transfer process (Figure 5A), which indicated that they are robust enough to be transferred to 96-well plates for subsequent drug screening applications. To further determine the cell viability quantitatively, an ATP-based cell viability assay was conducted. Compared with S0, the cell viability of both S-NS 005 and S-NS 01 from day 5 to day 11 was significantly higher, which was consistent with the live–dead staining results. However, no significant difference was observed between S-NS 005 and S-NS 01 (Figure 5B).

Compared with scaffold-free spheroids, the presence of nanoscaffolds leads to significantly higher cell viability, and no hypoxic area was observed. For spheroids without nanoscaffolds, the cells within the spheroid exhibited a compact arrangement, which could potentially lead to a hypoxic environment within the structure. This limitation in oxygen availability is a key factor that restricts the successful culture of spheroids and affects their overall viability and functionality. The high surface-volume ratio of our nanoscaffold could provide more attachment sites for cells.^54^ Furthermore, the incorporation of nanoscaffolds within the spheroids creates extra space, promoting the diffusion of oxygen and nutrients, which contributes to the overall viability and functionality of the spheroids, facilitating their growth and development.^46,54^

### Influence of Nanoscaffolds on the Recapitulation of In Vivo Functionality in HepG2 Spheroids

The primary indicators of liver-specific function include the continuous secretion of serum albumin and urea.^55,56^ Thus, the secretion levels of albumin and urea are used as a liver function test for liver spheroids. It has been shown that 2D cultured HepG2 cells will lose the ability of albumin and urea secretion rapidly.^57−59^ Therefore, we focused on a comparison of HepG2 spheroids with and without nanoscaffolds in this study.

**Figure 6:**
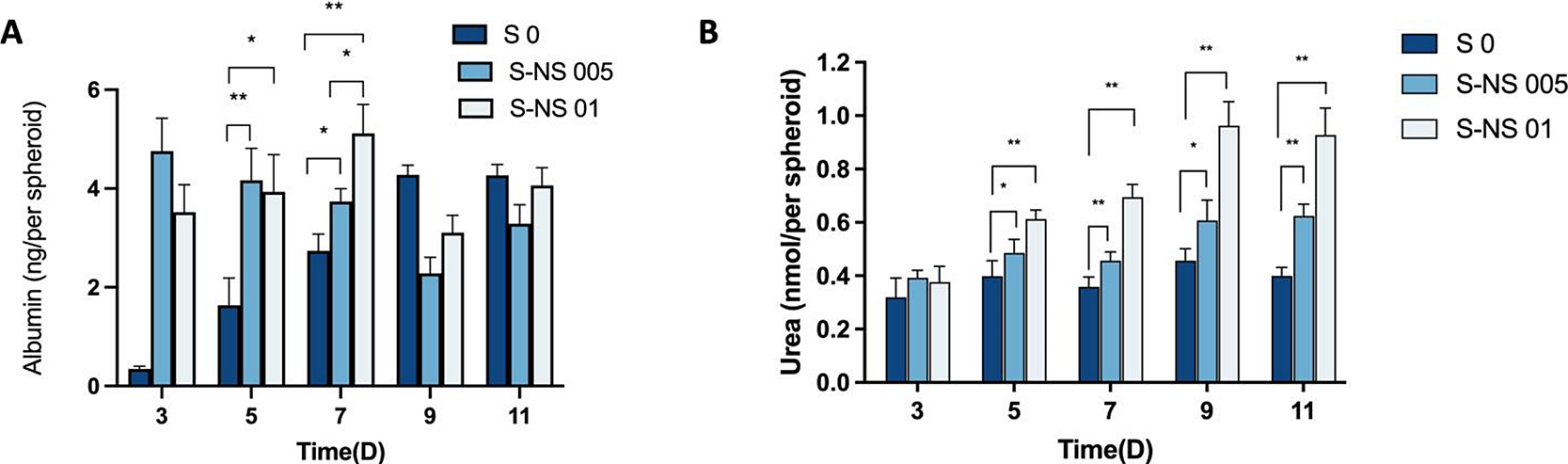
Influence of nanoscaffolds on the liver-specific functionality of spheroids. (A) Albumin secretion (*n* = 3); (B). Urea secretion (*n* = 3). Data are represented as mean ± standard error (* *p* < 0.05 ** *p* < 0.01).

As shown in Figure 6A, the albumin levels in S0 demonstrated a consistent increase over the course of 11 days. As for S-NS 01, there was an upward trend observed from day 3 to day 9, indicating an increase in albumin levels. A drop in albumin secretion was observed on day 9 in S-NS 01. A possible reason for this might be the fluctuation in cellular activity as similar observations have been reported in the literature.^60^ Interestingly, a decreasing trend in albumin secretion of S-NS 005 was observed. This may be due to the complexity of hepatocyte functional maintenance, where the functions could be influenced by biochemical or topological properties to some degree.^21^ In addition, S-NS 005 and S-NS 01 were significantly higher than S0 on days 3, 5, and 7. Finally, on day 7, S-NS 01 exhibited a more significant level of albumin than S-NS 005.

The synthesis of urea is another important liver-specific function of liver cells. Urea secretion of S 0 fluctuated from day 3 to day 11, reaching a peak on day 9 (Figure 6B). A similar trend was observed in S-NS 005 but with a higher secretion level than that of scaffold-free spheroids from day 5. For S-NS 01, a significantly higher urea level was observed from day 5 and the secretion kept increasing within 9 days. Compared with scaffold-free spheroid, the existence of nanoscaffolds promoted the secretion of urea after 5 days of culture.

**Figure 7:**
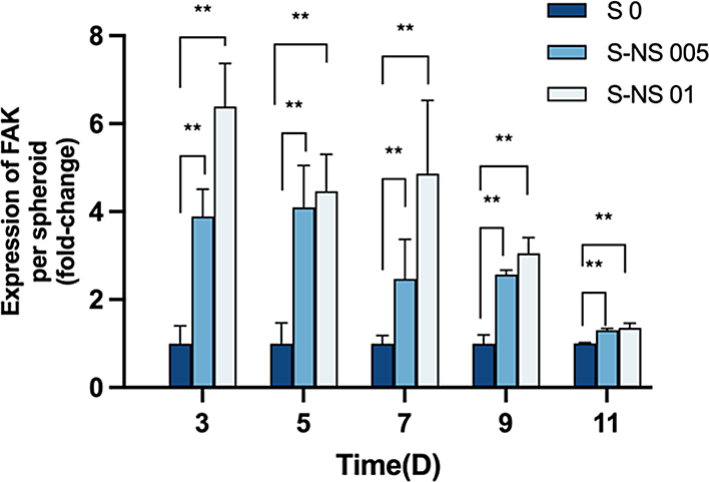
Fold change of FAK expression level between S 0, S-NS 005 and S-NS 01 (*n* = 3). Data are represented as mean ± standard error (* *p* < 0.05 ** *p* < 0.01).

All spheroids, with and without nanoscaffolds, showed sustained albumin and urea secretion over 11 days. Compared with scaffold-free spheroid, our findings show that the presence of the nanoscaffolds within the spheroid promoted the secretion of albumin and urea. This phenomenon could be due to the improved HepG2 cell-ECM interaction as a result of the nanoscaffolds, resulting in improved cell functionality. To verify this hypothesis, we then analyzed the expression of focal adhesion kinase (FAK), a molecular marker responsible for cell-ECM interactions. Integrins are the primary receptors that bind to the ECM and mediate cell-ECM interactions and FAK is the central mediator of integrin signaling transduction.^61^ As shown in Figure 7, the FAK levels of nanoscaffold-based spheroids (S-NS 005 and S-NS 01) were significantly higher than scaffold-free spheroids (S 0), especially during the formation phase of spheroids (the first 3 days). In addition, we found that the cells tended to bind on the nanoscaffolds first at the beginning of the formation process (Figure S3), which is consistent with the FAK results. These pieces of evidence preliminarily confirm our hypothesis that the presence of the nanoscaffolds could improve the cell-ECM interaction, but further research is still needed to explore the specific mechanisms.

Taking the findings into consideration, day 5 spheroids were chosen as optimal for drug screening experiments based on their favorable viability and functionality.

### Drug-Metabolising Enzyme Activity

**Figure 8:**
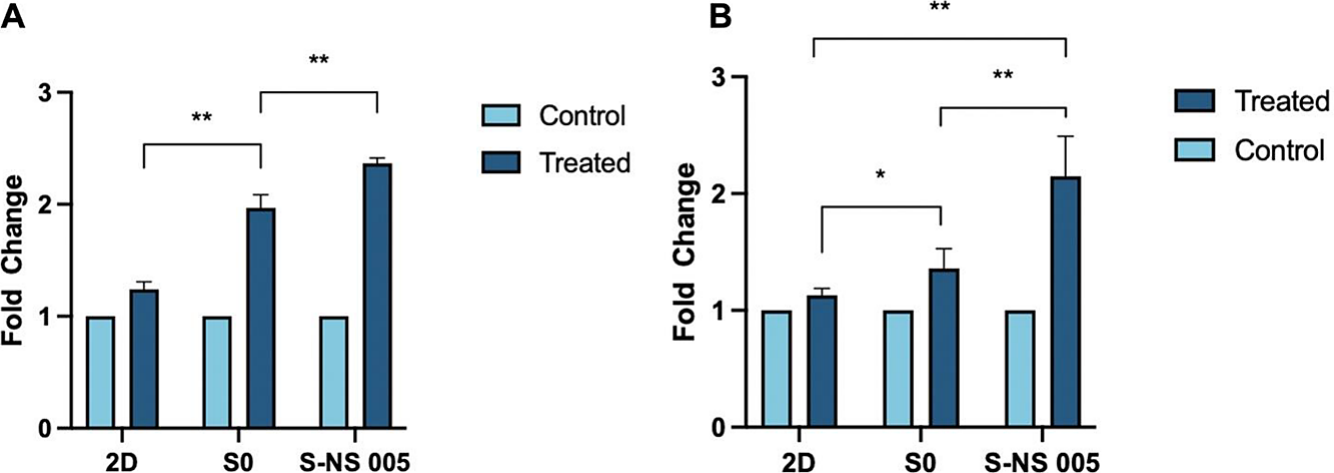
Fold change of enzyme activity of CYP 1A2 (A) and CYP 3A4 (B) after treatment with omeprazole and rifampicin for 72 h, respectively.

The liver is the major organ for drug metabolism and cytochrome P450 (CYP) is the most significant enzyme system responsible for the metabolism of the majority of drugs in the liver.^62^ Due to substantial interindividual variations in P450 and its susceptibility to various factors, obtaining information during the drug discovery stage about whether a new candidate drug is an effective CYP inducer is essential before selecting candidates for clinical development.^63^ Therefore, in vitro drug screening models (liver spheroids in this study) should possess the capability to assess the potential for CYP450 induction, as well as sensitivity to inducers and inhibitors of the enzyme. The activity of CYP 1A2 and CYP 3A4 was evaluated, and rifampicin and omeprazole were used as the inducers of CYP 3A4^64^ and CYP 1A2^65^ respectively. As shown in Figure 8, HepG2 cells exhibited minimal induction of CYP3A4 and CYP 1A2 following a 2D culture. In S0, the expressions of CYP3A4 and CYP1A2 increased by 1.94-fold and 1.36-fold, respectively. In S-NS 005, the inductions were more pronounced, with CYP3A4 and CYP1A2 increasing by 2.36-fold and 2.15-fold, respectively. The results indicated that our nanoscaffold-based spheroids are sensitive to enzyme inducers and are capable of being used as drug-screening tools.

### Acetaminophen Metabolism in HepG2 Spheroids

The prolonged hepatocyte viability and sustained functions in HepG2 spheroids can be beneficial for in vitro liver toxicity assays in comparison to 2D monolayer culture where cells quickly dedifferentiate and die.^66^ In order to further validate the predictive ability of drug metabolism in HepG2 spheroids, a dose–response toxicity analysis of acetaminophen was conducted and compared to that of HepG2 cells cultured as a 2D monolayer. Acetaminophen (APAP) is a nonopioid analgesic that is generally considered a safe medication when used at recommended therapeutic doses. The majority of intake APAP undergoes phase II metabolism in liver cells.^9^*N*-acetyl-p-benzoquinone imine (NAPQI) is recognized as the toxic reactive intermediate generated from APAP through the action of cytochrome P-450,^67^ especially in cases of acetaminophen overdose. NAPQI will lead to glutathione (GSH) depletion and cause hepatotoxicity by forming acetaminophen–protein adducts.

**Figure 9:**
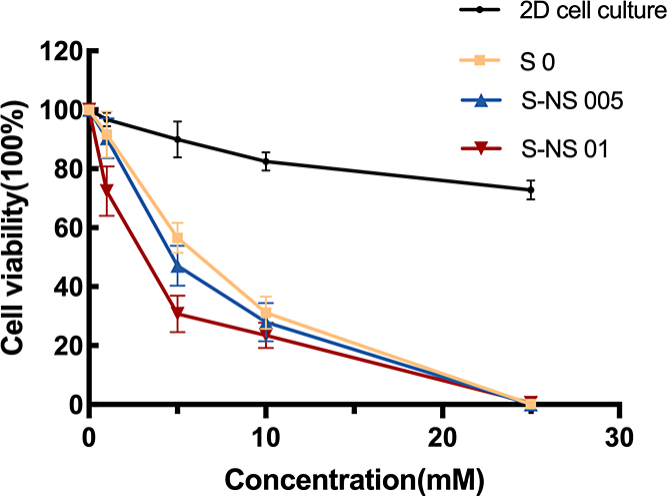
Acetaminophen metabolism. Data are represented as the mean ± standard error (*n* = 3).

The results showed a dose-dependent decrease in cell viability in both spheroids, with and without nanoscaffolds, while the 2D cell model was insensitive to APAP toxicity due to the lack of expression of metabolizing enzymes and thereby failed to detect liver toxicity (Figure 9). 3D cultured HepG2 cells (spheroids) have significantly enhanced enzyme expression,^68^ thereby providing a more accurate reflection of the metabolism and toxicity of APAP.

As shown in Table 1, the IC 50 value of acetaminophen in 2D cultured cells was 2469 μM while IC 50 values in spheroids were much lower (763.2, 692.8, 529.5 μM for S0, S-NS 005 and S-NS 01 respectively). The IC50 values in spheroids with nanoscaffolds were lower than in scaffold-free spheroids in our work. This suggests that the 3D spheroid model is more sensitive to hepatotoxins than standard 2D models and the presence of nanoscaffolds can further enhance the sensitivity. The IC50 ranges in our study were comparable to a previously published study on the drug sensitivity of primary human hepatocytes (PHH) spheroids, which are considered to be the “golden standard” for the in vitro DILI models, where the IC50 value of acetaminophen in PHH spheroids (927.7 μM) was significantly lower than that in monolayer culture (>20,000 μM).^70^

**Table 1:** IC50 Values of Acetaminophen in Different Models

cell culture model	IC 50 value (μM)
2D cell culture	2469
S0	763.2
S-NS 005	692.8
S-NS 01	529.5

## Conclusions

In this study, we fabricated hanging-drop HepG2 spheroids with PCL nanoscaffolds. Compared with scaffold-free spheroids, the cell viability was improved in our nanoscaffold-based spheroids, though no significant difference was observed from the increment of the nanoscaffold concentration added to the spheroids. In addition, we found that the spheroids with nanoscaffolds were able to synthesize and secrete more albumin and urea and exhibited enhanced drug-metabolizing enzyme activity than scaffold-free spheroids, which provided further evidence of liver-specific functionality in spheroids with nanoscaffolds. Toxicological analysis of a well-known hepatotoxin-acetaminophen indicated that the spheroid with a nanoscaffold can predict hepatotoxic potential with a higher sensitivity than standard 2D monolayer cultures and scaffold-free 3D spheroids. The results suggest that our scaffold-based spheroid models could significantly enhance the performance of liver spheroids. A possible reason is that the presence of the nanostructures could enhance the cell-ECM interaction. Notably, this nanoscaffold-based method has the potential to facilitate the in situ generation of spheroids from various cell types, offering a versatile platform that can be adapted to model different organs or diseases. By making suitable modifications to the nanoscaffolds or culture medium properties, our nanoscaffolds hold the potential to serve as a valuable tool in the field of tissue engineering.

## Materials and Methods

### Materials

PCL (average Mn 80,000) was purchased from Sigma-Aldrich (UK). Fetal bovine serum (FBS), Trypsin-EDTA solution, minimum essential medium eagle (MEME), MEM nonessential amino acid solution (NEAA), l-glutamine solution, and hexamethyldisilazane (HMDS) were purchased from Sigma-Aldrich (UK). Oxoid phosphate-buffered saline (PBS) tablets (Dulbecco A) Hoechst 33342 solution (20 mM), LIVE/DEAD cell imaging kit (488/570), fixative solution, AlamarBlue cell viability reagent, albumin human ELISA kit, FAK human ELISA kit, and coumarin 6 were purchased from Thermo-Fisher (UK). CellTiter-Glo 3D Cell Viability Assay reagent and P450-Glo CYP1A2 and 3A4 Assay were purchased from Promega (UK). The human hepatocellular carcinoma cell line (HepG2) was purchased from Abcam (UK).

### Preparation of PCL Nanoscaffolds

PCL polymer was dissolved with acetone at 60 °C for 2 h to reach a final concentration of 10% w/v. For the electrospinning process, the polymer solution was loaded into a 5 mL syringe and attached to a 19-gauge (inner diameter = 0.686 mm, outer diameter = 1.607 mm) needle. The syringe was then fixed onto a syringe pump at a distance of 15 cm from the grounded collector covered with aluminum foil. Electrospinning was performed at a voltage of 19 kV with a flow rate of 2 mL/h. The electrospinning experiments were performed at an ambient temperature of 20–22 °C and humidity of 40–50%. Short nanofibers were generated by mechanical grinding after freezing by liquid nitrogen. Thereafter, the short nanofibers were dried at room temperature for 24 h.

### Characterization of Nanoscaffolds

#### Scanning Electron Microscopy

The surface morphology of the electrospun fibrous membrane was examined by scanning electron microscopy (SEM) (Hitachi, Tokyo, Japan). Samples were coated with 10 nm gold and scanned at different magnifications to determine the morphology of the fibers. Images were analyzed using ImageJ digital analysis software to measure the diameters of the individual fibers. A total of 100 measurements were taken and used to obtain the average fiber diameter. A fiber diameter distribution histogram was plotted using GraphPad Prism 9.

#### Attenuated Total Reflection-Fourier Transform Infrared Spectroscopy (ATR-FTIR)

Characterization of raw materials and fibers was conducted using ATR-FTIR. Measurements were performed using a PerkinElmer spectrometer using the following parameters: resolution 4 cm^–1^; scan count was 22 scans (also for background) over 4000–500 cm^–1^ at ambient temperature (25 °C). Spectra were analyzed using CPU32 Main version 00.09.9951 and GraphPad Prism 9.

#### Porosity

The porosity of the nanofibrous scaffold was obtained by using the liquid displacement method. The samples were dropped into cell culture media and removed. The porosity of the nanofibrous scaffold was calculated using the following equation:
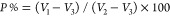


*V*_1_ - starting cell culture media volume

*V*_2_ - cell culture media volume immediately after the scaffold was added

*V*_3_ - remaining cell culture media volume after removing the scaffold

#### Cell Culture and Spheroid Generation

HepG2 cells were cultivated in MEME supplemented with 10% FBS, 1% NEAA, and 1% l-Glutamine. The HepG2 spheroids were generated by the hanging drop method as previously described.^15^ Briefly, trypsinized cells were pelleted at 1000 rpm for 3 min and then resuspended cells in a complete tissue culture medium. Cells were counted using a hemacytometer and the concentration was adjusted to 2.5 × 104 cells/mL. Twenty μL drops containing 500 cells were deposited onto the bottom of the Petri dish lid. Invert the lid was inserted into the PBS-filled bottom chamber and incubated at 37 °C/5% CO2/95% humidity. The cell culture medium was changed every 2 days.

For the generation of spheroids with nanoscaffolds, the nanoscaffolds were sterilized with a UV lamp for 12 h and then mixed with HepG2 cells at different nanofiber concentrations (0.005 and 0.01% w/v). The spheroids with nanofibers were generated and cultured with the same method as the ones without nanofibers.

The generation process of HepG2 spheroids was observed with a phase contrast microscope (Celena S, Logos Biosystems, UK) on different days, and the sizes were measured with image analysis software (ImageJ). Images of at least 20 spheroids in different views were taken, and the diameter of a spheroid was defined as the average length measured at two° intervals joining two outline points and passing through the centroid.

#### Distribution of Nanoscaffolds in Spheroids

To observe the distribution of nanofibers in spheroids, PCL nanofibre was labeled by adding a green fluorescent dye coumarin 6 to the PCL-acetone solution before electrospinning at a concentration of 1% (w/w).^69^ S-NS 005 were analyzed on day 5 after their generation by SEM for a detailed evaluation of their morphology and distribution of nanoscaffolds. For the preparation of SEM samples, first, the spheroids were collected and rinsed twice with PBS; then the spheroids were fixed at room temperature (RT) by Image-iT Fixative Solution for 45 min. Subsequently, the spheroids were dehydrated in an ascending ethanol series (70, 80, 90, and 100% ethanol) and then transferred to 50 and 100% HMDS for 15 min at RT. Finally, the spheroids were placed on a glass slide under the laboratory hood overnight, allowing the samples to dry completely. The glass slides were then transferred to conductive carbon adhesive tabs, sputtered with 10 nm, and analyzed with SEM. In addition, spheroids generated with coumarin-loaded nanoscaffolds were stained with 1 mg/mL Hoechst 33342 after 15 min of incubation at RT in the dark, followed by an extensive wash with PBS. Fluorescence images were obtained by a CELENA S Digital Imaging System (Logos Biosystems, UK) under excitation/emission wavelengths of 466/504 nm for coumarin 6 and 361/486 for Hoechst 33342.

#### 2D HepG2 Cell and HepG2 Spheroid Viability

The cell viability of 3D spheroids was examined by the CellTiter-Glo assay following the manufacturer’s protocol. For the cell imaging assay, spheroids were stained with a LIVE/DEAD Cell Imaging kit, and the stained spheroid samples were analyzed by light and fluorescence microscopy (Celena S) under the excitation/emission wavelengths of 488/515 nm for live cells (GFP) and 570/602 nm for dead cells (RFP).

The cell viability of 2D cultured HepG2 cells was determined by the Alamar blue method according to the manufacturer’s instructions. The absorbance of the reagent was measured using the absorbance plate reader at a wavelength of 570 nm.

#### Albumin and Urea Secretion Detection

Albumin and urea secretion levels of HepG2 spheroids with and without nanoscaffolds were quantified using an albumin human ELISA kit (Invitrogen) and urea assay kit (Abcam), respectively, according to the manufacturer’s protocol. The absorbance was read at a 450 nm wavelength.

#### Drug-Metabolising Enzyme Activity

Rifampicin and omeprazole were used as the inducers of CYP 3A4^64^ and CYP 1A2,^65^ respectively. For the inducement of CYP 3A4 and CYP 1A2, the 2D cells, S 0 and S-NS 005 (day 5) were treated with rifampicin (25 μM) and omeprazole (100 μM) for 72 h, respectively.

The drug-metabolism enzyme activity was then evaluated using P450-Glo CYP1A2 and 3A4 Assay kits according to the manufacturer’s protocol. Data were normalized to cell number measured by CellTiter-Glo assay.

#### Acetaminophen Metabolism

Acetaminophen (APAP) working solutions were prepared by diluting the 50 mM stock solution to give the following concentrations: 1, 5, 10, and 25 mM. On day 5, HepG2 spheroids with and without nanoscaffolds were transferred to a 96-well plate and exposed to various doses of APAP for 72 h at 37 °C. 2D cultured cells were used as the control group. Cell viability was measured by Alamar blue for 2D cultured cells and 3D Celltiter-Glo kit for 3D spheroids, respectively. IC50 values were calculated using GraphPad Prism.

#### Focal Adhesion Kinase (FAK) Level

Twenty spheroids with and without nanoscaffolds were collected on day 3, 5, 7, 9, 11. The FAK level was quantified using a FAK Human ELISA kit according to the manufacturer’s protocol. The absorbance of the reagent was measured using an absorbance plate reader at a wavelength of 450 nm. The data were shown as the fold change of the FAK level compared with spheroids without nanoscaffolds.

#### Statistical Analysis

Data are presented as the mean and standard deviation (SD) and are representative of two or more experiments. A student *t*-test was used to discern the statistical difference between the two groups. A probability value (*p*) of less than 0.05 was considered statistically significant. Graphs and statistical analysis were performed using GraphPad Prism 9 (GraphPad Software, San Diego, CA, USA).

## Supporting Information Available

Toxicity of PCL nanofiber on 2D HepG2 cells; HepG2 spheroids with different concentrations of PCL nanofibres on day 3; and generation of HepG2 spheroids with and without nanoscaffolds (PDF)

## References

[ref1] Singh V. K.; Seed T. M. How necessary are animal models for modern drug discovery?. Expert opinion on drug discovery 2021, 16 (12), 1391–1397. 10.1080/17460441.2021.1972255. 34455867

[ref2] Zhang D.; Luo G.; Ding X.; Lu C. Preclinical experimental models of drug metabolism and disposition in drug discovery and development. Acta Pharmaceutica Sinica B 2012, 2 (6), 549–561. 10.1016/j.apsb.2012.10.004.

[ref3] Han J. J.; Han J. J. FDA Modernization Act 2.0 allows for alternatives to animal testing; Wiley Online Library, 2023. 10.1111/aor.14503 36762462

[ref4] Freires I. A.; Sardi J. d. C. O.; de Castro R. D.; Rosalen P. L. Alternative animal and non-animal models for drug discovery and development: bonus or burden?. Pharm. Res. 2017, 34, 681–686. 10.1007/s11095-016-2069-z. 27858217

[ref5] Rice J. Animal models: Not close enough. Nature 2012, 484 (7393), S9–S9. 10.1038/nature11102. 22509510

[ref6] Fontoura J. C.; Viezzer C.; Dos Santos F. G.; Ligabue R. A.; Weinlich R.; Puga R. D.; Antonow D.; Severino P.; Bonorino C. Comparison of 2D and 3D cell culture models for cell growth, gene expression and drug resistance. Materials Science and Engineering: C 2020, 107, 110264 10.1016/j.msec.2019.110264. 31761183

[ref7] Kang S.-J.; Lee H.-M.; Park Y.-I.; Yi H.; Lee H.; So B.; Song J.-Y.; Kang H.-G. Chemically induced hepatotoxicity in human stem cell-induced hepatocytes compared with primary hepatocytes and HepG2. Cell biology and toxicology 2016, 32 (5), 403–417. 10.1007/s10565-016-9342-0. 27287938

[ref8] Baker B. M.; Chen C. S. Deconstructing the third dimension–how 3D culture microenvironments alter cellular cues. J. Cell Sci. 2012, 125 (13), 3015–3024. 10.1242/jcs.079509. 22797912 PMC3434846

[ref9] Ortega-Prieto A.; Skelton J.; Wai S.; Large E.; Lussignol M.; Vizcay-Barrena G.; Hughes D.; Fleck R.; Thursz M.; Catanese M. 3D microfluidic liver cultures as a physiological preclinical tool for hepatitis B virus infection. Nat. Commun. 2018, 9 (1), 682 10.1038/s41467-018-02969-8. 29445209 PMC5813240

[ref10] Nie Y.-Z.; Zheng Y.-W.; Miyakawa K.; Murata S.; Zhang R.-R.; Sekine K.; Ueno Y.; Takebe T.; Wakita T.; Ryo A. Recapitulation of hepatitis B virus–host interactions in liver organoids from human induced pluripotent stem cells. eBioMedicine 2018, 35, 114–123. 10.1016/j.ebiom.2018.08.014. 30120080 PMC6156717

[ref11] Friedrich J.; Seidel C.; Ebner R.; Kunz-Schughart L. A. Spheroid-based drug screen: considerations and practical approach. Nature protocols 2009, 4 (3), 309–324. 10.1038/nprot.2008.226. 19214182

[ref12] Lee G.; Kim H.; Park J. Y.; Kim G.; Han J.; Chung S.; Yang J. H.; Jeon J. S.; Woo D.-H.; Han C. Generation of uniform liver spheroids from human pluripotent stem cells for imaging-based drug toxicity analysis. Biomaterials 2021, 269, 120529 10.1016/j.biomaterials.2020.120529. 33257114

[ref13] Shao C.; Chi J.; Zhang H.; Fan Q.; Zhao Y.; Ye F. Development of cell spheroids by advanced technologies. Adv. Mater. Technol. 2020, 5 (9), 2000183 10.1002/admt.202000183.

[ref14] Sant S.; Johnston P. A. The production of 3D tumor spheroids for cancer drug discovery. Drug Discovery Today: Technologies 2017, 23, 27–36. 10.1016/j.ddtec.2017.03.002. 28647083 PMC5497458

[ref15] Foty R. A simple hanging drop cell culture protocol for generation of 3D spheroids. J. Vis. Exp. 2011, 51, e2720 10.3791/2720-v. PMC3197119 21587162

[ref16] Shah U.-K.; de Oliveira Mallia J.; Singh N.; Chapman K. E.; Doak S. H.; Jenkins G. J. A three-dimensional in vitro HepG2 cells liver spheroid model for genotoxicity studies. Mutat. Res./Genet. Toxicol. Environ. Mutagen. 2018, 825, 51–58. 10.1016/j.mrgentox.2017.12.005. 29307375

[ref17] Hurrell T.; Ellero A. A.; Masso Z. F.; Cromarty A. D. Characterization and reproducibility of HepG2 hanging drop spheroids toxicology in vitro. Toxicology in Vitro 2018, 50, 86–94. 10.1016/j.tiv.2018.02.013. 29476884

[ref18] Tibbitt M. W.; Anseth K. S. Hydrogels as extracellular matrix mimics for 3D cell culture. Biotechnology and bioengineering 2009, 103 (4), 655–663. 10.1002/bit.22361. 19472329 PMC2997742

[ref19] Saheli M.; Sepantafar M.; Pournasr B.; Farzaneh Z.; Vosough M.; Piryaei A.; Baharvand H. Three-dimensional liver-derived extracellular matrix hydrogel promotes liver organoids function. J. Cell Biochem 2018, 119 (6), 4320–4333. 10.1002/jcb.26622. 29247536

[ref20] Shin J.-Y.; Park J.; Jang H.-K.; Lee T.-J.; La W.-G.; Bhang S. H.; Kwon I. K.; Kwon O. H.; Kim B.-S. Efficient formation of cell spheroids using polymer nanofibers. Biotechnology letters 2012, 34 (5), 795–803. 10.1007/s10529-011-0836-9. 22207145

[ref21] Chua K.-N.; Lim W.-S.; Zhang P.; Lu H.; Wen J.; Ramakrishna S.; Leong K. W.; Mao H.-Q. Stable immobilization of rat hepatocyte spheroids on galactosylated nanofiber scaffold. Biomaterials 2005, 26 (15), 2537–2547. 10.1016/j.biomaterials.2004.07.040. 15585256

[ref22] Zhang K.; Bai X.; Yuan Z.; Cao X.; Jiao X.; Qin Y.; Wen Y.; Zhang X. Cellular nanofiber structure with secretory activity-promoting characteristics for multicellular spheroid formation and hair follicle regeneration. ACS Appl. Mater. Interfaces 2020, 12 (7), 7931–7941. 10.1021/acsami.9b21125. 32003218

[ref23] Mironov V.; Khesuani Y. D.; Bulanova E. A.; Koudan E. V.; Parfenov V. A.; Knyazeva A. D.; Mitryashkin A. N.; Replyanski N.; Kasyanov V. A.; DAS F. P. Patterning of tissue spheroids biofabricated from human fibroblasts on the surface of electrospun polyurethane matrix using 3D bioprinter. Int. J. Bioprint. 2016, 2 (1), 45–52. 10.18063/IJB.2016.01.007.

[ref24] Cox C. R.; Lynch S.; Goldring C.; Sharma P. Current perspective: 3D spheroid models utilizing human-based cells for investigating metabolism-dependent drug-induced liver injury. Front. Med. Technol. 2020, 2, 611913 10.3389/fmedt.2020.611913. 35047893 PMC8757888

[ref25] Onakpoya I. J.; Heneghan C. J.; Aronson J. K. Post-marketing withdrawal of 462 medicinal products because of adverse drug reactions: a systematic review of the world literature. BMC Med. 2016, 14 (1), 10 10.1186/s12916-016-0553-2. 26843061 PMC4740994

[ref26] Mishra S. K.; Singh P.; Rath S. K. A study of toxicity and differential gene expression in murine liver following exposure to anti-malarial drugs: amodiaquine and sulphadoxine-pyrimethamine. Malar. J. 2011, 10 (1), 109 10.1186/1475-2875-10-109. 21529379 PMC3112449

[ref27] Stevens J. L.; Baker T. K. The future of drug safety testing: expanding the view and narrowing the focus. Drug discovery today 2009, 14 (3–4), 162–167. 10.1016/j.drudis.2008.11.009. 19100337

[ref28] Bell C. C.; Hendriks D. F.; Moro S. M.; Ellis E.; Walsh J.; Renblom A.; Fredriksson Puigvert L.; Dankers A. C.; Jacobs F.; Snoeys J. Characterization of primary human hepatocyte spheroids as a model system for drug-induced liver injury, liver function and disease. Sci. Rep. 2016, 6 (1), 25187 10.1038/srep25187. 27143246 PMC4855186

[ref29] Basharat A.; Rollison H. E.; Williams D. P.; Ivanov D. P. HepG2 (C3A) spheroids show higher sensitivity compared to HepaRG spheroids for drug-induced liver injury (DILI). Toxicol. Appl. Pharmacol. 2020, 408, 115279 10.1016/j.taap.2020.115279. 33068618

[ref30] Lauschke V. M.; Hendriks D. F.; Bell C. C.; Andersson T. B.; Ingelman-Sundberg M. Novel 3D culture systems for studies of human liver function and assessments of the hepatotoxicity of drugs and drug candidates. Chemical research in toxicology 2016, 29 (12), 1936–1955. 10.1021/acs.chemrestox.6b00150. 27661221

[ref31] Choi J. M.; Oh S. J.; Lee S. Y.; Im J. H.; Oh J. M.; Ryu C. S.; Kwak H. C.; Lee J.-Y.; Kang K. W.; Kim S. K. HepG2 cells as an in vitro model for evaluation of cytochrome P450 induction by xenobiotics. Archives of pharmacal research 2015, 38, 691–704. 10.1007/s12272-014-0502-6. 25336106

[ref32] Donato M. T.; Tolosa L.; Gómez-Lechón M. J. Culture and functional characterization of human hepatoma HepG2 cells. Protocols in in vitro hepatocyte research 2015, 1250, 77–93. 10.1007/978-1-4939-2074-7_5. 26272135

[ref33] Ramaiahgari S. C.; Den Braver M. W.; Herpers B.; Terpstra V.; Commandeur J. N.; van de Water B.; Price L. S. A 3D in vitro model of differentiated HepG2 cell spheroids with improved liver-like properties for repeated dose high-throughput toxicity studies. Arch. Toxicol. 2014, 88, 1083–1095. 10.1007/s00204-014-1215-9. 24599296

[ref34] Gerets H.; Tilmant K.; Gerin B.; Chanteux H.; Depelchin B.; Dhalluin S.; Atienzar F. Characterization of primary human hepatocytes, HepG2 cells, and HepaRG cells at the mRNA level and CYP activity in response to inducers and their predictivity for the detection of human hepatotoxins. Cell biology and toxicology 2012, 28, 69–87. 10.1007/s10565-011-9208-4. 22258563 PMC3303072

[ref35] Štampar M.; Breznik B.; Filipič M.; Žegura B. Characterization of in vitro 3D cell model developed from human hepatocellular carcinoma (HepG2) Cell Line. Cells 2020, 9 (12), 2557 10.3390/cells9122557. 33260628 PMC7759933

[ref36] Mansouri M.; Beemer S.; Kothapalli C. R.; Rhoades T.; Fodor P. S.; Das D.; Leipzig N. D. Generation of oxygenating fluorinated methacrylamide chitosan microparticles to increase cell survival and function in large liver spheroids. ACS Appl. Mater. Interfaces 2022, 14 (4), 4899–4913. 10.1021/acsami.1c19962. 35060707

[ref37] Pazhanimala S. K.; Vllasaliu D.; Raimi-Abraham B. T. Electrospun nanometer to micrometer scale biomimetic synthetic membrane scaffolds in drug delivery and tissue engineering: a review. Applied Sciences 2019, 9 (5), 910 10.3390/app9050910.

[ref38] Malikmammadov E.; Tanir T. E.; Kiziltay A.; Hasirci V.; Hasirci N. PCL and PCL-based materials in biomedical applications. Journal of Biomaterials science, Polymer edition 2018, 29 (7–9), 863–893. 10.1080/09205063.2017.1394711. 29053081

[ref39] Pazhanimala S. K.; Vllasaliu D.; Raimi-Abraham B. T. Engineering biomimetic gelatin based nanostructures as synthetic substrates for cell culture. Applied Sciences 2019, 9 (8), 1583 10.3390/app9081583.

[ref40] Chong E. J.; Phan T. T.; Lim I. J.; Zhang Y.; Bay B. H.; Ramakrishna S.; Lim C. T. Evaluation of electrospun PCL/gelatin nanofibrous scaffold for wound healing and layered dermal reconstitution. Acta biomaterialia 2007, 3 (3), 321–330. 10.1016/j.actbio.2007.01.002. 17321811

[ref41] Jalali S.; Kruppke I.; Enghardt S.; Wiesmann H. P.; Kruppke B. Silica Nanofibers with Enhanced Wettability and Mechanical Strength for Bone Tissue Engineering: Electrospinning without Polymer Carrier and Subsequent Heat Treatment. Macromol. Mater. Eng. 2024, 309 (1), 2300169 10.1002/mame.202300169.

[ref42] Pelipenko J.; Kocbek P.; Kristl J. Nanofiber diameter as a critical parameter affecting skin cell response. European Journal of Pharmaceutical Sciences 2015, 66, 29–35. 10.1016/j.ejps.2014.09.022. 25301202

[ref43] Tian F.; Hosseinkhani H.; Hosseinkhani M.; Khademhosseini A.; Yokoyama Y.; Estrada G. G.; Kobayashi H. Quantitative analysis of cell adhesion on aligned micro-and nanofibers. J. Biomed. Mater. Res. A 2008, 84 (2), 291–299. 10.1002/jbm.a.31304. 17607759

[ref44] Wang J.; Ye R.; Wei Y.; Wang H.; Xu X.; Zhang F.; Qu J.; Zuo B.; Zhang H. The effects of electrospun TSF nanofiber diameter and alignment on neuronal differentiation of human embryonic stem cells. J. Biomed. Mater. Res. A 2012, 100 (3), 632–645. 10.1002/jbm.a.33291. 22213384

[ref45] Wei J.; Lei D.; Chen M.; Ran P.; Li X. Engineering HepG2 spheroids with injectable fiber fragments as predictable models for drug metabolism and tumor infiltration. Journal of Biomedical Materials Research Part B: Applied Biomaterials 2020, 108 (8), 3331–3344. 10.1002/jbm.b.34669. 32627303

[ref46] Lee J.; Lee S.; Kim S. M.; Shin H. Size-controlled human adipose-derived stem cell spheroids hybridized with single-segmented nanofibers and their effect on viability and stem cell differentiation. Biomater. Res. 2021, 25 (1), 14 10.1186/s40824-021-00215-9. 33902733 PMC8074457

[ref47] Liu Y.; Zhang L.; Wei J.; Yan S.; Yu J.; Li X. Promoting hepatocyte spheroid formation and functions by coculture with fibroblasts on micropatterned electrospun fibrous scaffolds. J. Mater. Chem. B 2014, 2 (20), 3029–3040. 10.1039/c3tb21779e. 32261678

[ref48] Feng Z.-Q.; Chu X.-H.; Huang N.-P.; Leach M. K.; Wang G.; Wang Y.-C.; Ding Y.-T.; Gu Z.-Z. Rat hepatocyte aggregate formation on discrete aligned nanofibers of type-I collagen-coated poly (L-lactic acid). Biomaterials 2010, 31 (13), 3604–3612. 10.1016/j.biomaterials.2010.01.080. 20149442

[ref49] Reed C. R.; Han L.; Andrady A.; Caballero M.; Jack M. C.; Collins J. B.; Saba S. C.; Loboa E. G.; Cairns B. A.; van Aalst J. A. Composite tissue engineering on polycaprolactone nanofiber scaffolds. Annals of plastic surgery 2009, 62 (5), 505–512. 10.1097/SAP.0b013e31818e48bf. 19387150

[ref50] Hirschhaeuser F.; Menne H.; Dittfeld C.; West J.; Mueller-Klieser W.; Kunz-Schughart L. A. Multicellular tumor spheroids: an underestimated tool is catching up again. Journal of biotechnology 2010, 148 (1), 3–15. 10.1016/j.jbiotec.2010.01.012. 20097238

[ref51] Anada T.; Fukuda J.; Sai Y.; Suzuki O. An oxygen-permeable spheroid culture system for the prevention of central hypoxia and necrosis of spheroids. Biomaterials 2012, 33 (33), 8430–8441. 10.1016/j.biomaterials.2012.08.040. 22940219

[ref52] Lee J.; Lilly G. D.; Doty R. C.; Podsiadlo P.; Kotov N. A. In vitro toxicity testing of nanoparticles in 3D cell culture. Small 2009, 5 (10), 1213–1221. 10.1002/smll.200801788. 19263430

[ref53] Bell C. C.; Dankers A. C.; Lauschke V. M.; Sison-Young R.; Jenkins R.; Rowe C.; Goldring C. E.; Park K.; Regan S. L.; Walker T. Comparison of hepatic 2D sandwich cultures and 3D spheroids for long-term toxicity applications: a multicenter study. Toxicol. Sci. 2018, 162 (2), 655–666. 10.1093/toxsci/kfx289. 29329425 PMC5888952

[ref54] Ahmad T.; Lee J.; Shin Y. M.; Shin H. J.; Perikamana S. K. M.; Park S. H.; Kim S. W.; Shin H. Hybrid-spheroids incorporating ECM like engineered fragmented fibers potentiate stem cell function by improved cell/cell and cell/ECM interactions. Acta Biomater. 2017, 64, 161–175. 10.1016/j.actbio.2017.10.022. 29037892

[ref55] Papageorgopoulos C.; Caldwell K.; Shackleton C.; Schweingrubber H.; Hellerstein M. K. Measuring protein synthesis by mass isotopomer distribution analysis (MIDA). Analytical biochemistry 1999, 267 (1), 1–16. 10.1006/abio.1998.2958. 9918649

[ref56] Li R.; Liu J.; Ma J.; Sun X.; Wang Y.; Yan J.; Yu Q.; Diao J.; Yang C.; Reid L. M. Fibrinogen improves liver function via promoting cell aggregation and fibronectin assembly in hepatic spheroids. Biomaterials 2022, 280, 121266 10.1016/j.biomaterials.2021.121266. 34875515

[ref57] Gaskell H.; Sharma P.; Colley H. E.; Murdoch C.; Williams D. P.; Webb S. D. Characterization of a functional C3A liver spheroid model. Toxicology research 2016, 5 (4), 1053–1065. 10.1039/C6TX00101G. 27746894 PMC5047049

[ref58] Kojima N.; Matsuo T.; Sakai Y. Rapid hepatic cell attachment onto biodegradable polymer surfaces without toxicity using an avidin–biotin binding system. Biomaterials 2006, 27 (28), 4904–4910. 10.1016/j.biomaterials.2006.05.026. 16759691

[ref59] Chu Q.; Zhao Y.; Shi X.; Han W.; Zhang Y.; Zheng X.; Zhu J. In vivo-like 3-D model for sodium nitrite-and acrylamide-induced hepatotoxicity tests utilizing HepG2 cells entrapped in micro-hollow fibers. Sci. Rep. 2017, 7 (1), 14837 10.1038/s41598-017-13147-z. 29093461 PMC5665964

[ref60] Bhise N. S.; Manoharan V.; Massa S.; Tamayol A.; Ghaderi M.; Miscuglio M.; Lang Q.; Zhang Y. S.; Shin S. R.; Calzone G. A liver-on-a-chip platform with bioprinted hepatic spheroids. Biofabrication 2016, 8 (1), 014101 10.1088/1758-5090/8/1/014101. 26756674

[ref61] Smyrek I.; Mathew B.; Fischer S. C.; Lissek S. M.; Becker S.; Stelzer E. H. E-cadherin, actin, microtubules and FAK dominate different spheroid formation phases and important elements of tissue integrity. Biol. Open 2019, 8 (1), bio037051 10.1242/bio.037051. 30578251 PMC6361217

[ref62] Meyer U. A. Overview of enzymes of drug metabolism. Journal of pharmacokinetics and biopharmaceutics 1996, 24, 449–459. 10.1007/BF02353473. 9131484

[ref63] Lin J. H. CYP induction-mediated drug interactions: in vitro assessment and clinical implications. Pharm. Res. 2006, 23, 1089–1116. 10.1007/s11095-006-0277-7. 16718615

[ref64] Yamashita F.; Sasa Y.; Yoshida S.; Hisaka A.; Asai Y.; Kitano H.; Hashida M.; Suzuki H. Modeling of rifampicin-induced CYP3A4 activation dynamics for the prediction of clinical drug-drug interactions from in vitro data. PloS one 2013, 8 (9), e70330 10.1371/journal.pone.0070330. 24086247 PMC3782498

[ref65] Han X. M.; Ouyang D. S.; Chen X. P.; Shu Y.; Jiang C. H.; Tan Z. R.; Zhou H. H. Inducibility of CYP1A2 by omeprazole in vivo related to the genetic polymorphism of CYP1A2. British journal of clinical pharmacology 2002, 54 (5), 540–543. 10.1046/j.1365-2125.2002.01686.x. 12445035 PMC1874453

[ref66] Mizoi K.; Arakawa H.; Yano K.; Koyama S.; Kojima H.; Ogihara T. Utility of three-dimensional cultures of primary human hepatocytes (spheroids) as pharmacokinetic models. Biomedicines 2020, 8 (10), 374 10.3390/biomedicines8100374. 32977664 PMC7598599

[ref67] Harvison P. J.; Guengerich F. P.; Rashed M. S.; Nelson S. D. Cytochrome P-450 isozyme selectivity in the oxidation of acetaminophen. Chemical research in toxicology 1988, 1 (1), 47–52. 10.1021/tx00001a009. 2979711

[ref68] Štampar M.; Tomc J.; Filipič M.; Žegura B. Development of in vitro 3D cell model from hepatocellular carcinoma (HepG2) cell line and its application for genotoxicity testing. Arch. Toxicol. 2019, 93, 3321–3333. 10.1007/s00204-019-02576-6. 31542801

[ref69] Jiang J.; Chen S.; Wang H.; Carlson M. A.; Gombart A. F.; Xie J. CO2-expanded nanofiber scaffolds maintain activity of encapsulated bioactive materials and promote cellular infiltration and positive host response. Acta biomaterialia 2018, 68, 237–248. 10.1016/j.actbio.2017.12.018. 29269334 PMC5803415

[ref70] Li F.; Cao L.; Parikh S.; Zuo R. Three-dimensional spheroids with primary human liver cells and differential roles of Kupffer cells in drug-induced liver injury. J. Pharm. Sci. 2020, 109 (6), 1912–1923. 10.1016/j.xphs.2020.02.021. 32145211

