# Supplementary material for: In Situ Self-Assembling Liver Spheroids with Synthetic Nanoscaffolds for Preclinical Drug Screening Applications
Source: ACS Appl Mater Interfaces. 2024 May 14;16(20):25610–21. doi: 10.1021/acsami.3c17384 (PMC11129140; doi:10.1021/acsami.3c17384)
Supplement: Supplementary file 1 — am3c17384_si_001.pdf [file am3c17384_si_001.pdf]

Supporting Information

In-Situ Self-Assembling Liver Spheroids with Synthetic Nanoscaffolds for Preclinical Drug Screening Applications

Lina Wu,[1] Driton Vllasaliu[1], Qi Cui[1] and Bahijja Tolulope Raimi-Abraham[1]*

*** Corresponding Author:** Dr Bahijja Raimi-Abraham, *Bahijja.Raimi-Abraham@kcl.ac.uk*

King's College London, Faculty of Life Sciences and Medicine, School of Cancer and Pharmaceutical Sciences, Institute of Pharmaceutical Science, Franklin-Wilkins Building, 150 Stamford Street, London SE1 9NH, United Kingdom

Influence of PCL nanofibre on HepG2 cell viability and proliferation

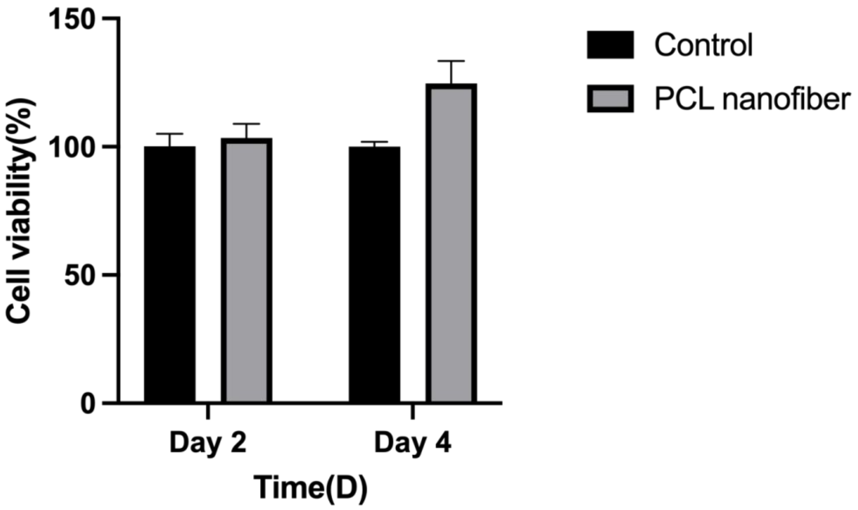

Figure S1. Toxicity of PCL nanofibre on 2D HepG2 cells.

Influence of Nanoscaffold Concentration on Spheroid Formation

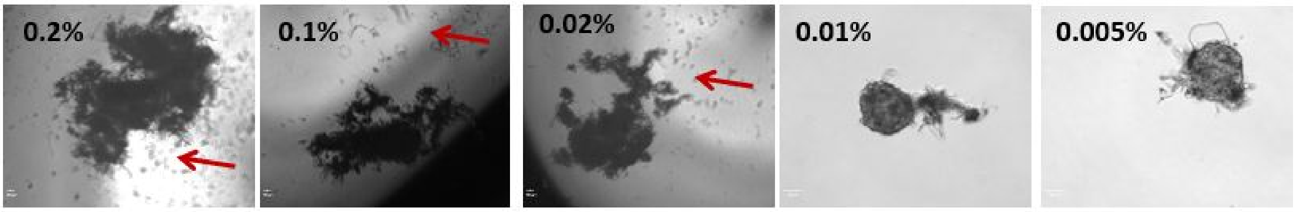

Figure S2. HepG2 Spheroids with different concentrations of PCL nanofibres on Day 3. Scale bar: 50 µm. Objective:10X.

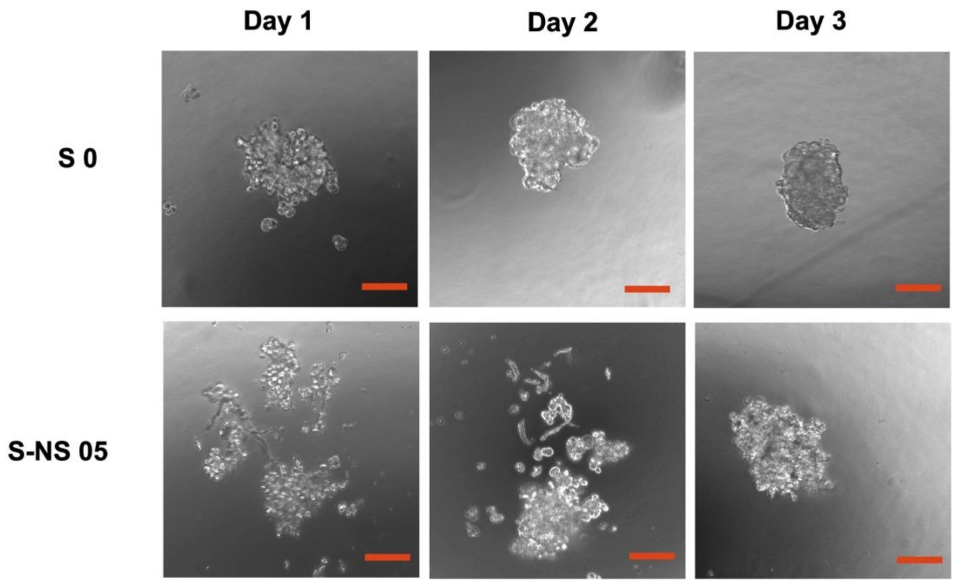

Figure S3. Generation of HepG2 Spheroids with and without nanoscaffolds. Scale bar: 100 µm.
